# HIV-Associated Pseudoaneurysms: A Comprehensive Review

**DOI:** 10.7759/cureus.72076

**Published:** 2024-10-21

**Authors:** Umar Magid, Hanifa Ismail, Maheen Zahid, Khatab W Ahmad, Muhammad Ahmad, Hashir Nazir, Abdullah K Alassiri, Osman S Ahmed, Amr T Bakhit, Tehseen Raza

**Affiliations:** 1 Medicine, Trakia University, Stara Zagora, BGR; 2 Medicine, International European University, Kyiv, UKR; 3 Medicine, King Edward Medical University, Lahore, PAK; 4 Medicine, University of Sharjah, Sharjah, ARE; 5 Medicine, Faculty of Medicine, King Abdulaziz University, Jeddah, SAU; 6 Medicine, Faculty of Medicine, University of Khartoum, Khartoum, SDN; 7 Medicine, University of Medical Sciences and Technology, Khartoum, SDN

**Keywords:** arterial disease, artery pseudoaneurysm, hiv aids, pathogenesis, vascular complications

## Abstract

A pseudoaneurysm (PSA) is a contained vascular rupture that typically occurs following catheterization, at the anastomotic site between a native artery and a synthetic graft, post-trauma, or as a result of infection. It is characterized by a hematoma surrounded by tissue, often emerging as a complication of invasive arterial interventions. In patients with HIV/AIDS, PSAs can develop due to vessel wall disruption caused by chronic inflammation, opportunistic infections (such as cytomegalovirus or tuberculosis), or the direct effects of the virus, leading to abnormal blood flow into a chamber confined by adjacent tissue. The clinical presentation of PSAs varies based on their size and location. Diagnosis can be achieved through ultrasonography with color Doppler, contrast-enhanced computed tomography (CT), magnetic resonance angiography (MRA), or digital subtraction angiography (DSA). Treatment modalities include surgery, ultrasound-guided compression, thrombin injection, and endovascular techniques. This review discusses the pathophysiology, histology, diagnosis, and therapeutic options for HIV-related PSAs. Additionally, risk factors and rare complications associated with PSAs are explored in detail.

## Introduction and background

Human immunodeficiency virus (HIV) infection remains one of the most significant global public health concerns. HIV was first identified in young men who have sex with men (MSM) in the United States in 1981, who presented with opportunistic infections such as pneumocystis pneumonia, and AIDS-related conditions like Kaposi's sarcoma, alongside severe immunodeficiency [1]. As an immunosuppressant virus, HIV leads to acquired immunodeficiency syndrome (AIDS), causing a variety of opportunistic infections and affecting multiple organ systems, including the liver, gastrointestinal tract, kidneys, and vascular system. HIV-associated vascular complications can manifest as arteritis, perivasculitis, vasculitis, and aneurysms [2]. The virus responsible for HIV was discovered in 1983, and it was confirmed that it causes AIDS. A key indicator of AIDS is a CD4 count below 200 cells/µL, with advanced AIDS characterized by a severely weakened immune system that predisposes patients to opportunistic infections. HIV vasculopathy, first recognized in 1987, presents as spontaneous arteriovenous fistulas, aneurysmal disease, and arterial occlusive conditions. Symptomatic vasculitis affects approximately 1% of individuals living with HIV [3].

The widespread use of potent antiretroviral therapy (ART) has significantly reduced HIV-related mortality and morbidity. However, as the lifespan of people with HIV has increased, so too has the prevalence of chronic complications related to the virus [4]. HIV-positive individuals are more prone to developing pseudoaneurysms (PSAs), potentially due to direct viral replication in the arterial walls, which may result in vasculitis. Studies have demonstrated a correlation between PSA formation in HIV-positive patients and inflammation of the vasa vasorum, as well as decreased CD4 counts [5].

PSAs have been documented in surgical literature since 1946. Their incidence has increased in recent decades due to the growing number of endovascular interventions and intravenous drug use. A PSA occurs when a tear in the vessel wall leads to the formation of a periarterial hematoma. If left untreated, PSAs can progress, potentially rupturing and resulting in life-threatening hemorrhage [6]. A pulsating encapsulated hematoma in communication with the arterial lumen is characteristic of a PSA. The terms "false aneurysms," "pulsatile hematomas," and "communicating hematomas" are often used interchangeably [7]. Unlike true aneurysms, which are enclosed within all three layers of the vessel wall, PSAs are not fully bound by these layers, distinguishing them from true aneurysms [8]. Aneurysmal disease in HIV-positive patients often presents with systemic clinical symptoms, low CD4 counts, and multifocal lesions at unusual sites, typically affecting younger individuals and occurring in advanced stages of HIV [9]. The pathophysiology underlying these PSAs is multifaceted, primarily driven by the immunocompromised state induced by HIV infection, which predisposes individuals to various infections and vascular abnormalities. HIV can lead to vasculitis, a condition characterized by inflammation of blood vessels, which is a critical factor in the formation of PSAs. This vasculitis may result from direct viral invasion of the vascular endothelium or from immune-mediated processes that cause endothelial damage and subsequent weakening of the vessel wall [10,11]. The incidence of PSAs in HIV-positive patients is notably higher compared to the general population. For instance, studies have shown that in HIV patients, carotid artery PSAs can account for up to 44% of cases, a stark contrast to the 10% observed in non-infected individuals [12]. This increased prevalence is attributed to the combination of HIV-related vasculopathy and the heightened risk of infections that can lead to mycotic PSAs. Mycotic PSAs, which are caused by infectious agents, are particularly concerning in HIV patients due to their compromised immune systems, making them more susceptible to severe infections that can compromise vascular integrity [5,13].

In addition to the direct effects of HIV, other factors such as co-infections (e.g., tuberculosis) and lifestyle choices (e.g., intravenous drug use) further complicate the clinical picture. For example, intravenous drug users are at an elevated risk for developing PSAs due to the potential for skin infections and direct vascular trauma from needle use [14]. Management of HIV-associated PSAs poses unique challenges. Surgical interventions, including open repair and endovascular techniques, have been employed, but outcomes can be variable due to the underlying immunocompromised state of patients [8,15]. The complexity of treatment is compounded by the need to address not only the PSA itself but also the underlying HIV infection and any co-existing conditions that may contribute to vascular complications [15].

This review focuses on an understudied aspect of HIV-related vascular complications: PSAs. It aims to explore the pathogenesis, clinical presentation, and management strategies for HIV-associated PSAs, with a particular emphasis on the unique features of HIV-related vasculitis. Additionally, this review examines the epidemiology, risk factors, and clinical implications of PSAs, highlighting the importance of prompt diagnosis and intervention to prevent severe complications, such as rupture and hemorrhage. By providing an overview of current treatment strategies and their outcomes, this review seeks to expand the understanding of PSAs in the context of HIV.

## Review

Overview of HIV and its cardiovascular impact

In 2023, more than 1.3 million new cases of HIV infection were reported globally, contributing to the estimated 39.9 million people living with the virus [16]. While many HIV-positive individuals are under 50 years old, the use of ART has resulted in an increasing number of older patients. In the United States, over 45% of HIV patients are now older than 50, and by 2030, it is projected that 25% of HIV patients on ART will be over 65, with the median age rising to 53 [17]. The intersection of HIV infection, ART, and traditional cardiovascular disease (CVD) risk factors has contributed to a significant rise in atherosclerotic CVD among HIV-infected individuals, leading to an increase in CVD-related morbidity and mortality [18].

The elevated incidence of CVD in HIV/AIDS patients arises from a complex interplay of factors, including HIV-specific elements such as immune activation, chronic inflammation, and persistent infection. Additionally, conventional cardiovascular risk factors such as tobacco and illicit drug use, obesity, dyslipidemia, diabetes mellitus, and hypertension further exacerbate the risk. ART itself and disparities in healthcare access also contribute significantly. The highest burden of multimorbidity, including conditions like diabetes, hypertension, obesity, CVD, and renal failure, is seen in HIV-positive Black women and men [19]. Even after controlling for factors such as blood lipids, blood pressure, and tobacco use, CVD prevalence is found to be 50% higher in HIV-positive individuals. This suggests that both the viral infection and ART contribute to vascular endothelial dysfunction and, consequently, CVD [20].

Despite the use of ART, HIV remains in the body, predisposing patients to opportunistic infections, chronic immune activation, and ongoing inflammation. Common co-infections in HIV-positive individuals include herpes simplex virus, varicella-zoster virus, and cytomegalovirus (CMV), the latter being linked to elevated CMV antibody titers and chronic inflammation [17]. One of the primary theories regarding the immunological abnormalities in HIV patients posits that HIV directly infects and destroys CD4 T cells (helper T cells), leading to widespread immune dysfunction. The immunological disturbances associated with HIV are as complex as the immune system itself [21].

The mechanism underlying HIV-related vasculitis is thought to involve several factors beyond direct invasion of blood vessels and immune responses. Pathogen-associated molecular pattern molecules produced by the gut microbiota activate immune cells, leading to chronic inflammation. This persistent inflammation damages the endothelium, triggering the production of oxidized low-density lipoprotein (LDL). The interaction between oxidized LDL and immune cells exacerbates endothelial injury and perpetuates inflammation, contributing to the development of atherosclerosis. In HIV-related vasculitis, the inflammatory response predominantly affects the tunica adventitia, which may result in aneurysms and vascular obstruction [10]. Studies have demonstrated that discontinuing highly active ART (HAART) for AIDS increases the incidence of this condition. HIV-related vasculitis often impacts peripheral arteries, with the carotid and superficial femoral arteries being particularly vulnerable to aneurysm formation and arterial blockage. Additionally, aneurysms in HIV-positive individuals frequently occur at multiple sites or in locations atypical for atherosclerotic aneurysms [22]. Despite ongoing debate over the use of intravenous repair (IVR) therapy for ruptured aneurysms in HIV-positive patients, there are relatively few studies addressing the management of large artery aneurysms in this population.

Pathophysiology of PSAs

A PSA is defined as a chronic abnormality of the native arterial wall that results in the pulsatile extravasation of arterial blood outside the artery, contained by the surrounding soft tissues [23]. PSAs occur when the integrity of the arterial wall is compromised due to trauma, inflammation, or iatrogenic factors such as drainage, percutaneous biopsy, or surgery. Blood escapes into the surrounding tissues and forms a perfused sac that communicates with the arterial lumen under sustained arterial pressure. The media, adventitia, or adjacent soft tissue structures around the injured vessel serve as barriers to contain the perfused sac [24].

PSAs can be categorized as iatrogenic, traumatic, anastomotic, or infectious based on their etiology. While blunt trauma can cause PSAs, they are more commonly the result of penetrating injuries such as stab wounds, gunshots, or repeated arterial punctures, particularly in intravenous drug users [7]. Iatrogenic causes, including post-catheterization complications and surgical procedures, are also common [25]. Angiographic catheterization is the most frequent iatrogenic cause of PSA formation [26]. Other contributing factors include radiation exposure, connective tissue disorders, and, in rare cases, spontaneous occurrence [27]. A PSA forms when injury to the vascular wall leads to an encapsulated hematoma in communication with the ruptured artery. Unlike a true aneurysm, which involves all three layers of the arterial wall, a PSA is a collection of blood outside the vessel, contained by the surrounding tissue . True aneurysms make up the majority of arterial lesions, while PSAs account for less than 1%. In rare instances, PSAs may arise spontaneously due to atherosclerotic disease or congenital defects [28].

Epidemiology and risk factors

In contrast to the general population, where 1-3% of individuals in the age group of 50-60 years are affected, people with chronic HIV infection, atherosclerotic risk factors, or HAART-related metabolic syndrome may experience clinically significant peripheral arterial disease approximately two decades earlier [9]. Research by Qudeer et al. reported a 52% occurrence of PSAs in HIV-positive patients, with 92% of cases involving femoral and popliteal aneurysms and 8% affecting the brachial artery [29].

The exact mechanism by which HIV leads to PSAs remains unclear. However, it is hypothesized that immune complexes formed in response to HIV weaken the arterial wall. Immune activation in HIV-positive individuals results in the persistent activation of monocytes, macrophages, and CD8+ T-cells, leading to the release of pro-inflammatory and pro-fibrotic cytokines such as interleukin (IL)-1β, IL-6, soluble tumor necrosis factor α receptor 1 (sTNF-αR1), sTNF-αR2, monocyte chemotactic factor (CCL2), soluble CD163, soluble CD14, and intercellular adhesion molecule-1 (ICAM-1) [30]. These cytokines drive chronic inflammation, which plays a crucial role in vascular endothelial dysfunction, hypercoagulation, vascular thrombosis, collagen hyperproliferation, left ventricular remodeling, and hyperlipidemia, all of which contribute to CVD [17].

Vascular wall inflammation can result in aneurysm formation, which increases the risk of vessel rupture and subsequent PSA development. Calabrese et al. suggested that ulcerated atherosclerotic plaques in HIV-positive patients may be prone to aneurysms due to HIV infection, though this has yet to be proven [31]. Microscopically, HIV-associated vasculopathy exhibits features consistent with leukocytoclastic vasculitis, particularly affecting the vasa vasorum [32]. Histologically, HIV-related aneurysms demonstrate unique characteristics compared to degenerative and infectious aneurysms. These include fragmentation, duplication, and calcification of the internal elastic lamina, along with fibromuscular hyperplasia in the intima. The media often shows fibrosis, thickening, smooth muscle loss, and elastic fiber fragmentation, while the adventitia may exhibit mixed inflammation, hemosiderin-laden macrophages, collagen fragmentation, and leukocytoclastic vasculitis of the vasa vasorum [33].

The large vessels, such as the carotid arteries and lower limb arteries, are most commonly affected in HIV-associated vasculopathy [15]. The prevalence of HIV-related carotid aneurysms has been increasing due to the high incidence of HIV in the population. It is estimated that HIV-related vasculopathy accounts for 25% of all HIV-related aneurysms, including those affecting the carotid artery [34]. Risk factors for developing PSAs include the use of dual platelet inhibition and additional anticoagulants (triple therapy). Some studies suggest that using sheaths with French gauge sizes between six and eight may increase the risk of PSA formation, although this finding is not universally supported. Other established risk factors include advanced age, female sex, diabetes, cardiovascular conditions (particularly arterial hypertension and peripheral artery occlusive disease), and abnormal body weight (either overweight or underweight) [35].

Diagnosis of HIV-associated PSAs

Duplex sonography is the gold standard for diagnosing PSAs. This imaging modality provides a non-invasive, cost-effective method for assessing blood flow velocity without the need for contrast agents. Duplex ultrasound has demonstrated a sensitivity of up to 94% and a specificity of up to 97% [8]. Alternatives to duplex sonography include CT scans, magnetic resonance angiography (MRA), and digital subtraction angiography (DSA), though these methods pose risks such as radiation exposure and the need for contrast agents [8]. These techniques are particularly useful in complex cases or when the PSA originates in the retroperitoneal space. Although DSA is less commonly used due to its potential complications, it offers both diagnostic and therapeutic value [35].

Ultrasound findings of HIV-associated aneurysms typically include vessel wall thickening near the aneurysm, with a visible defect in the vessel wall. Hyperechoic areas, which may resemble arterial wall calcification or dense plaques, are most pronounced at the aneurysm site and diminish proximally and distally. A large anechoic cavity containing a thrombus with pulsatile blood flow is often seen adjacent to the affected vessels on duplex ultrasound [32]. The characteristic "yin and yang" sign, indicative of a PSA, is observed on color flow imaging. This sign, also referred to as the "to-and-fro" curve, reflects the systolic and diastolic blood flow in and out of the PSA and is visible in Doppler waveform analysis [36].

On non-contrast-enhanced CT scans, a PSA appears as a well-defined, rounded structure with smooth walls and low attenuation, emerging from the side of the parent artery. Following intravenous contrast administration, the pseudoaneurysmal sac fills, matching the attenuation of the parent artery. A thrombus within the sac appears as a filling defect. The adjacent donor artery and the neck connecting the sac to the artery become visible, facilitating evaluation. Contrast extravasation may indicate a rupture of the pseudoaneurysmal sac. CT angiography offers 95.1% sensitivity and 98.7% specificity [25].

DSA provides high spatial resolution, making it particularly useful in cases of ruptured PSAs where life-threatening hemorrhage requires immediate intervention. MRA can also be valuable in situations where patients have an allergic reaction to iodinated contrast media or when DSA is not desirable, although MRA is often less practical due to its higher cost [25,37].

Management and treatment approaches

Ultrasound-Guided Thrombin Injection Therapy

Ultrasound-guided thrombin injection therapy was first introduced by Cope and Zeit in 1986 [38]. Thrombin, a tissue adhesive used to stop bleeding and promote wound healing, is derived from bovine or synthetic sources. Under ultrasound guidance, thrombin is injected into the PSA. To position the needle accurately, 0.9% sodium chloride (NaCl) is used. The needle, connected to a syringe containing thrombin and sodium chloride via a three-way stopcock, is inserted into the PSA cavity while aspirating. The correct position is confirmed by the injection and aspiration of NaCl, after which thrombin is administered once the localization is verified [39]. Ultrasound-guided thrombin injection therapy boasts a success rate of 92-100%, but it carries risks, including thrombin migration beyond the PSA neck into the bloodstream, which can cause thrombus formation and limb ischemia [40]. Other potential complications include immune responses to bovine thrombin, leading to anaphylaxis, particularly in patients who have undergone repeated treatments for recurrent PSAs. Infections, accidental punctures, and arterial thrombosis are additional risks [35].

Endovascular Treatment

In endovascular treatment, a surgical incision is made distal to the aneurysm to accommodate the size of the introducer sheath needed for advancing the stent graft. Care is taken to prevent the stent from contacting the vessel wall directly. A transducer and imaging catheter are used to guide the procedure, and an angiogram is performed after positioning the guidewire distal to the aneurysm. The ultrasound catheter is advanced over the guidewire to capture images, allowing for proper stent apposition and determining the stent graft diameter [41]. Endovascular methods have demonstrated high success rates, with over 90% success and less than 8% perioperative morbidity within the first 30 days post treatment for non-traumatic aneurysms [9]. The advantages of endovascular therapy include its minimally invasive nature, distant access, avoidance of tissue plane dissection, reduced blood loss, quicker patient mobilization, shorter hospital stays, and lower rates of mortality, morbidity, and complications, such as neurological and venous issues [42].

In HIV-positive patients undergoing endovascular treatment, antiplatelet therapy is critical for optimal outcomes, though there is ongoing debate regarding the indications, duration, type, and dosage of these agents [43]. A study of 24 patients who underwent endovascular treatment for HIV-associated aneurysms reported postoperative complications in only three cases. Two cases involved stent occlusion in carotid artery aneurysms, while the third, following popliteal stent insertion, experienced late stent thrombosis after six months, leading to limb amputation [9]. Stent graft infection, though rare, is a serious complication that requires lifelong antibiotic prophylaxis in affected patients [44].

Surgical Approaches

The most common surgical techniques for treating PSAs include suturing the arterial wall defect after hematoma evacuation or performing patch angioplasty [25]. Studies suggest that open surgery is generally preferred in patients younger than 50 years of age [45]. In HIV patients with aneurysmal disease, open reconstructions have demonstrated mortality and morbidity rates of approximately 15% and 33%, respectively [33]. Several factors influence surgical outcomes in HIV-positive patients, including immunological status (CD4 T-cell count), presence of opportunistic infections, white blood cell count, hematocrit levels, nutritional status (albumin levels), type of surgery, and hygiene [46]. A study by Padachy and Robbs, involving 21 patients of which 16 underwent open surgical intervention, reported one case each of surgical wound sepsis, stroke, postoperative death, and three cases of new cranial nerve deficits [47]. Surgical treatment is contraindicated in patients with poor cardiac reserve due to the increased risks associated with invasive procedures. Moreover, open surgery carries a longer recovery time and presents a higher risk of complications such as bleeding, infections, and atrial fibrillation [48].

Complications and prognosis of HIV-associated PSAs

The prognosis for PSAs is generally favorable, though it varies depending on the location. Conservative therapy, combined with surveillance of femoral PSAs less than 3 cm in size, has resolution rates ranging from 50% to 100%. However, in patients receiving dual antiplatelet therapy, success rates are significantly lower, with failure rates reaching up to 44% [49]. Ultrasound-guided thrombin injection for femoral PSAs caused by vascular access has a high success rate of 97-100%, even in patients on anticoagulant or antiplatelet therapy. Surgical correction of PSAs typically involves simple interrupted sutures or patch angioplasty for large arterial defects. In cases of hematomas, drainage may be performed, and complications such as blood loss, infection, or nerve injury can be managed during surgery. Repeat treatments can be considered if the initial attempt fails [50].

Endovascular repair of visceral PSAs has shown promising technical success in small case series, although research in this area remains limited. Open repair and ligation, however, are considered more durable, particularly in younger patients [51]. Untreated cardiac PSAs have a nearly 45% chance of rupture and a 50% mortality rate in patients managed with medical therapy alone. Thoracic endovascular aortic repair (TEVAR) for traumatic aortic PSAs has demonstrated a 100% technical success rate, with a 2.4% rate of device-related complications and conversion to open procedures. Additionally, a delayed revascularization rate of 6% was observed following coverage of the left subclavian artery [52].

PSAs can rupture as they grow, potentially leading to hemodynamic shock. As the PSA expands, it may compress surrounding nerves and vessels, causing ischemic, sensory, and thrombotic complications. Intraoperative and postoperative complication rates for PSAs are as high as 21%, including bleeding, thrombosis, wound healing disorders, infections, edema, lymphatic fistulas, and permanent neuralgia [35]. PSAs of any type can lead to distal embolization, rupture, hemorrhage, and even death. Rupture of femoral PSAs into the retroperitoneal space can cause severe bleeding, which may go unnoticed initially and result in fatal outcomes. Distal embolization occurs in up to 2% of patients following ultrasound-guided thrombin injection, though only a small number of these cases require further intervention [53].

Future perspectives

The future perspectives on HIV-associated PSAs encompass advancements in diagnostic techniques, treatment modalities, and an understanding of the underlying pathophysiological mechanisms. As the population of people living with HIV continues to grow, the incidence of vascular complications, including PSAs, is expected to rise, necessitating enhanced clinical awareness and innovative management strategies. One promising area of development is the improvement in imaging techniques, which have significantly enhanced the detection of PSAs. Advances in radiological imaging, such as high-resolution ultrasound and CT angiography, allow for better visualization of vascular anomalies, leading to earlier diagnosis and intervention [54]. Enhanced imaging capabilities can facilitate the identification of asymptomatic PSAs, which may otherwise go unnoticed until they present with complications. Future studies should focus on standardizing imaging protocols to optimize the detection and management of PSAs in HIV-infected patients [55]. The management strategies for PSAs differ significantly between HIV-positive and HIV-negative patients. In HIV-negative individuals, conventional approaches such as surgical repair or standard endovascular techniques are often sufficient due to their generally robust immune systems. However, in HIV-positive patients, the risk of complications is heightened, necessitating a more tailored approach. For instance, the use of certain embolic agents like thrombin may be contraindicated in patients with a history of allergic reactions, which can be more prevalent in those with HIV [56]. Furthermore, the presence of co-infections or other comorbidities in HIV patients can complicate the clinical picture, making it imperative to utilize advanced imaging techniques and multimodal treatment strategies to ensure optimal outcomes [10].

In terms of treatment, the shift towards less invasive endovascular techniques is noteworthy. Endovascular approaches, such as ultrasound-guided thrombin injection and the use of covered stents, have shown promising results in managing PSAs with reduced morbidity compared to traditional surgical methods [40]. The ongoing development of novel materials and techniques, such as the use of biodegradable stents and advanced embolization agents such as n-butyl cyanoacrylate (NBCA), Onyx, and ethylene-vinyl alcohol (EVOH) copolymer-based non-adhesive liquid embolic agents (NALEAs), may further improve outcomes for patients with HIV-associated PSAs [41,57,58]. Additionally, research into the long-term efficacy and safety of these minimally invasive procedures is essential to establish best practices for managing this complex patient population [59].

Moreover, understanding the pathophysiology of HIV-associated vascular complications is crucial for developing targeted therapies. Research has indicated that HIV-related vasculopathy may lead to arterial wall weakening, increasing the risk of PSA formation [60]. Future investigations should explore the molecular mechanisms underlying this process, potentially identifying biomarkers that could predict the risk of vascular complications in people with HIV. Furthermore, the role of co-infections, particularly tuberculosis, in exacerbating vascular issues in HIV patients warrants further exploration, as this could inform comprehensive management strategies [60]. Lastly, the integration of lifestyle interventions, such as exercise, may offer additional benefits in preventing vascular aging and improving overall health in people with HIV. Studies suggest that regular physical activity could mitigate some of the vascular dysfunction associated with HIV, potentially reducing the incidence of PSAs and other cardiovascular complications [61]. Future research should aim to elucidate the relationship between lifestyle factors and vascular health in this population, paving the way for holistic treatment approaches that encompass both medical and lifestyle interventions.

## Conclusions

HIV-associated pseudoaneurysms represent a significant and complex vascular complication, particularly in the context of long-term ART and chronic immune activation. While advancements in diagnostic techniques, such as duplex ultrasound, CT angiography, and DSA, have improved the ability to identify and manage DSAs, optimal treatment approaches remain a topic of ongoing research. Ultrasound-guided thrombin injection and endovascular techniques offer high success rates with relatively low complications, but surgical intervention may still be necessary in specific cases, especially in younger patients or those with severe disease. Understanding the underlying mechanisms of HIV-related vasculopathy, including immune system dysregulation and inflammation, is crucial for managing PSAs effectively.

Given the potential for life-threatening complications such as rupture, hemorrhage, and distal embolization, early detection and prompt intervention are critical. Further studies are needed to evaluate the long-term outcomes of different treatment modalities, particularly in the HIV-positive population, where comorbidities and immune status may influence prognosis. This review underscores the importance of personalized treatment strategies, continued research, and vigilance in monitoring HIV-positive patients for vascular complications to improve patient outcomes and reduce morbidity and mortality associated with PSAs.

## References

[1] (1981). Kaposi's sarcoma and pneumocystis pneumonia among homosexual men--New York City and California. MMWR Morb Mortal Wkly Rep.

[2] Tenório GO, Silvestre JM, Sardinha WE (2013). Pseudoaneurysms in association with human immunodeficiency virus infection: report of two cases [Article in Spanish]. J Vasc Bras.

[3] Mulaudzi TV (2009). HIV-associated vasculopathy. Contin Med Educ.

[4] Kumar P, Arendt C, Martin S, Al Soufi S, DeLeuw P, Nagel E, Puntmann VO (2023). Multimodality imaging in HIV-associated cardiovascular complications: a comprehensive review. Int J Environ Res Public Health.

[5] Nair R, Abdool-Carrim A, Chetty R, Robbs J (1999). Arterial aneurysms in patients infected with human immunodeficiency virus: a distinct clinicopathology entity?. J Vasc Surg.

[6] Rabinovich Y, Klemperer LN, Levi Y, Rubinstein C, Rosenthal E, Sheick-Yousif B (2023). An out of the box treatment for an infected pseudoaneurysm: deep to superficial femoral artery transposition. J Vasc Surg Cases Innov Tech.

[7] Sapkota R, Sapkota S, Shrestha K, Rajbhandari N, Shrestha U (2011). Management of pseudoaneurysms in IV drug users. J Inst Med.

[8] Alexandre K (2022). Endovascular management of a large femoral pseudoaneurysm: a case report and literary review. Cureus.

[9] Pillay B, Ramdial PK, Naidoo DP, Sartorius B, Singh D (2016). Endovascular therapy for large vessel vasculopathy in HIV-infected patients. Eur J Vasc Endovasc Surg.

[10] Iseki Y, Fujii M, Nishina D (2021). A case of human immunodeficiency virus-positive patient diagnosed during the treatment of right internal iliac pseudoaneurysm. Ann Vasc Dis.

[11] Pillay B, Ramdial PK, Naidoo DP (2015). HIV-associated large-vessel vasculopathy: a review of the current and emerging clinicopathological spectrum in vascular surgical practice. Cardiovasc J Afr.

[12] Fraund-Cremer S, Bernd R, Cremer J, Rusch R (2022). Progressive cervical tumour in an HIV-patient: giant pseudoaneurysm of the carotid artery: a case report. Eur Heart J-Case Rep.

[13] Barber L, Jordan A, Douglas R (2022). Fatal epistaxis in a case of common variable immunodeficiency: a case report and review of the literature. Clin Case Rep.

[14] Shrestha KR, Gurung D, Khanal N, Shrestha UK (2020). Femoral pseudoaneurysm in IV drug abusers: single-center study experience. J Nepal Health Res Counc.

[15] Saraf R, Narang A, Kardile M, Udmale P (2018). Endovascular treatment of HIV-associated spontaneous common carotid artery pseudoaneurysm in a case of miliary and CNS tuberculosis. Br J Neurosurg.

[16] (2024). UNAIDS: Global HIV & AIDS statistics — fact sheet. https://www.unaids.org/en/resources/fact-sheet.

[17] Henning RJ, Greene JN (2023). The epidemiology, mechanisms, diagnosis and treatment of cardiovascular disease in adult patients with HIV. Am J Cardiovasc Dis.

[18] Sarkar S, Brown TT (2021). CROI 2021: metabolic and other complications of HIV infection or COVID-19. Top Antivir Med.

[19] Ghosn J, Taiwo B, Seedat S, Autran B, Katlama C (2018). HIV. Lancet Lond Engl.

[20] Kovacs L, Kress TC, Belin de Chantemèle EJ (2022). HIV, combination antiretroviral therapy, and vascular diseases in men and women. Basic Transl Sci.

[21] Edelman AS, Zolla-Pazner S (1989). AIDS: a syndrome of immune dysregulation, dysfunction, and deficiency. FASEB J.

[22] Robbs JV, Paruk N (2010). Management of HIV vasculopathy - a South African experience. Eur J Vasc Endovasc Surg.

[23] Jaff MR (2002). Pseudoaneurysms. Curr Treat Options Cardiovasc Med.

[24] Saad NE, Saad WE, Davies MG, Waldman DL, Fultz PJ, Rubens DJ (2005). Pseudoaneurysms and the role of minimally invasive techniques in their management. Radiographics.

[25] Keeling AN, McGrath FP, Lee MJ (2009). Interventional radiology in the diagnosis, management, and follow-up of pseudoaneurysms. Cardiovasc Intervent Radiol.

[26] Kalapatapu VR, Shelton KR, Ali AT, Moursi MM, Eidt JF (2008). Pseudoaneurysm: a review. Curr Treat Options Cardiovasc Med.

[27] Zheng Y, Lu Z, Shen J, Xu F (2020). Intracranial pseudoaneurysms: evaluation and management. Front Neurol.

[28] Khandelwal P, Akkara F, Dhupar V, Louis A (2018). Traumatic pseudoaneurysm of the superficial temporal artery. Natl J Maxillofac Surg.

[29] Qudeer MA, Naqi SA, Sarwar MZ, Mujahid HA (2021). Intravenous drug abusers presenting with pseudoaneurysm and other surgical complications in Pakistan. J Pak Med Assoc.

[30] Durstenfeld MS, Hsue PY (2021). Mechanisms and primary prevention of atherosclerotic cardiovascular disease among people living with HIV. Curr Opin HIV AIDS.

[31] Calabrese LH, Estes M, Yen-Lieberman B, Proffitt MR, Tubbs R, Fishleder AJ, Levin KH (1989). Systemic vasculitis in association with human immunodeficiency virus infection. Arthritis Rheum.

[32] Woolgar JD, Ray R, Maharaj K, Robbs JV (2002). Colour Doppler and grey scale ultrasound features of HIV-related vascular aneurysms. Br J Radiol.

[33] Botes K, Van Marle J (2007). Surgical intervention for HIV related vascular disease. Eur J Vasc Endovasc Surg.

[34] Roos M, Butler I (2016). Extracranial internal carotid artery pseudoaneurysm in a two-year-old child: case report. J Laryngol Otol.

[35] Peters S, Braun-Dullaeus R, Herold J (2018). Pseudoaneurysm. Hamostaseologie.

[36] Abu-Yousef MM, Wiese JA, Shamma AR (1988). The "to-and-fro" sign: duplex Doppler evidence of femoral artery pseudoaneurysm. AJR Am J Roentgenol.

[37] Nastri MV, Baptista LP, Baroni RH (2004). Gadolinium-enhanced three-dimensional MR angiography of Takayasu arteritis. Radiographics.

[38] Cope C, Zeit R (1986). Coagulation of aneurysms by direct percutaneous thrombin injection. AJR Am J Roentgenol.

[39] La Perna L, Olin JW, Goines D, Childs MB, Ouriel K (2000). Ultrasound-guided thrombin injection for the treatment of postcatheterization pseudoaneurysms. Circulation.

[40] Kang SS, Labropoulos N, Mansour MA, Michelini M, Filliung D, Baubly MP, Baker WH (2000). Expanded indications for ultrasound-guided thrombin injection of pseudoaneurysms. J Vasc Surg.

[41] Ryan JM, Dumbleton SA, Doherty J, Smith TP (2003). Technical innovation. Using a covered stent (wallgraft) to treat pseudoaneurysms of dialysis grafts and fistulas. AJR Am J Roentgenol.

[42] Martinakis VG, Dalainas I, Katsikas VC, Xiromeritis K (2013). Endovascular treatment of carotid injury. Eur Rev Med Pharmacol Sci.

[43] Matas M, Domínguez González JM, Montull E (2013). Antiplatelet therapy in endovascular surgery: the RENDOVASC study. Ann Vasc Surg.

[44] Kritpracha B, Premprabha D, Sungsiri J, Tantarattanapong W, Rookkapan S, Juntarapatin P (2011). Endovascular therapy for infected aortic aneurysms. J Vasc Surg.

[45] Morbi A, Gohel MS, Hamady M, Cheshire NJ, Bicknell CD (2012). Lower-limb ischemia in the young patient: management strategies in an endovascular era. Ann Vasc Surg.

[46] Binderow SR, Cavallo RJ, Freed J (1993). Laboratory parameters as predictors of operative outcome after major abdominal surgery in AIDS- and HIV-infected patients. Am Surg.

[47] Padayachy V, Robbs JV (2012). Carotid artery aneurysms in patients with human immunodeficiency virus. J Vasc Surg.

[48] Piffaretti G, Mariscalco G, Tozzi M, Rivolta N, Castelli P, Sala A (2011). Predictive factors of complications after surgical repair of iatrogenic femoral pseudoaneurysms. World J Surg.

[49] Stone PA, Martinez M, Thompson SN, Masinter D, Campbell JE, Campbell Ii JR, AbuRahma AF (2016). Ten-year experience of vascular surgeon management of iatrogenic pseudoaneurysms: do anticoagulant and/or antiplatelet medications matter?. Ann Vasc Surg.

[50] Stone PA, Campbell JE, AbuRahma AF (2014). Femoral pseudoaneurysms after percutaneous access. J Vasc Surg.

[51] Regus S, Lang W (2016). Rupture risk and etiology of visceral artery aneurysms and pseudoaneurysms: a single-center experience. Vasc Endovascular Surg.

[52] Azizzadeh A, Ray HM, Dubose JJ (2014). Outcomes of endovascular repair for patients with blunt traumatic aortic injury. J Trauma Acute Care Surg.

[53] Rivera PA, Dattilo JB (2024). Pseudoaneurysm. StatPearls [Internet].

[54] Jin L, Fitzgerald A (2021). Delayed diagnosis of dorsal scapular artery pseudoaneurysm following blunt chest trauma. Trauma Case Rep.

[55] Murray KD, Singh MV, Zhuang Y (2020). Pathomechanisms of HIV-associated cerebral small vessel disease: a comprehensive clinical and neuroimaging protocol and analysis pipeline. Front Neurol.

[56] Zabicki B, Limphaibool N, Holstad MJ, Juszkat R (2018). Endovascular management of pancreatitis-related pseudoaneurysms: a review of techniques. PLoS One.

[57] Larner B, Maingard J, Ren Y (2019). Endovascular treatment of a hepatic artery pseudoaneurysm using a novel pericardium covered stent. J Med Imaging Radiat Oncol.

[58] Minici R, Guerriero P, Fontana F (2023). Endovascular treatment of visceral artery pseudoaneurysms with ethylene-vinyl alcohol (EVOH) copolymer-based non-adhesive liquid embolic agents (NALEAs). Medicina (Kaunas).

[59] Karsonovich TW, Hawkins JC, Gordhan A (2019). Traumatic pseudoaneurysm of the ascending cervical artery treated with N-butyl cyanoacrylate embolization: a case report and review of the literature. Cureus.

[60] Crisp J, Ahmad M, Crockett S, Mohamed A, Hamady M, Bernstein O, Shalhoub J (2024). Spontaneous bilateral superficial femoral artery pseudoaneurysms and a unilateral posterior tibial artery aneurysm in an immunocompromised patient. Clin Case Rep.

[61] Jones R, Robinson AT, Beach LB (2024). Exercise to prevent accelerated vascular aging in people living with HIV. Circ Res.

